# Transnational Health and Self-care Experiences of Japanese Women who have taken Oral Contraceptives in South Korea, including Over-the-counter Access: Insights from Semi-structured Interviews

**DOI:** 10.1007/s41649-024-00293-6

**Published:** 2024-04-11

**Authors:** Seongeun Kang, Kazuto Kato

**Affiliations:** 1 Department of Biomedical Ethics and Public Policy, Graduate School of Medicine, Osaka University, Osaka, Japan; 2 Department of Medical Humanities and Social Sciences, Yonsei University College of Medicine, Seoul, South Korea

**Keywords:** Transnational health-seeking, Oral contraceptives, Over-the-counter access, Medical pluralism, Self-care, Global perspectives

## Abstract

In an increasingly globalized world, the accessibility of healthcare and medication has expanded beyond local healthcare systems and national borders. This study aims to investigate the transnational health and self-care experiences of 11 Japanese women who have resided in South Korea for a minimum of six months and have utilized oral contraceptives, including those that were acquired over-the-counter (OTC). Data were gathered through semi-structured interviews and analyzed by utilizing the NVivo software. The analysis yielded three significant thematic categories, namely (1) experiences and perceptions of obtaining and utilizing contraceptive pills, including OTC access; (2) individual and social perceptions of pills and their accessibility in Japan, insights from actual users; and (3) enhancing pill accessibility, transnational health and self-care experiences and perspectives. Participants acknowledged that oral contraceptives are a global product and experienced communication challenges with healthcare providers as a result of differing understandings of these medications. Additionally, this study identified transnational strategies, such as purchasing an adequate supply of pills just before departure and seeking pills from local families or acquaintances. This study not only highlights the implications of clinical care for transnational patients but also underscores their critical global perspectives on access to oral contraceptives. Furthermore, it proposes two models for improving accessibility within the Japanese healthcare system, even in prescription-only contexts, by introducing OTC options.

## Introduction

Healthcare systems and access to medicines vary significantly depending on the country and region. Globalization, characterized by the transnational movement of people, goods, services, capital, and information, has significantly diversified the healthcare experiences for patients and users across international borders (Durham and Blondell 2017; Bell et al. 2015; Glinos et al. 2010). This phenomenon encompasses various financial, institutional, cultural, and emotional factors that influence the healthcare choices of patients. Prior research has explored topics in this realm, such as medical tourism, the health of foreign and migrants’ health, and the use of surrogacy from a transnational approach (Gray et al. 2023; Şekercan et al. 2021; Arvidsson et al. 2018; Carone et al. 2017; Horton 2013; Lee et al. 2010; Rivera et al. 2009; Krause 2008). More specifically, some researchers have explained the phenomenon of patients’ transnational medical use and migrants’ utilization of homeland healthcare as a concept of transnational health-seeking (Gray et al. 2023; Keller and Alishio-Caballero 2021; Gilbert et al. 2019; Sun 2014; Gideon 2011).

Studies on the utilization and accessibility of oral contraceptives have been conducted, particularly in the border regions of the USA and Mexico. These studies predominantly focus on user satisfaction with OTC access, considering factors, such as cost and convenience, and have highlighted improvements in access to healthcare services, clinics, and medicines as perceived by patients and users (White et al. 2013; Hopkins et al. 2012; Potter et al. 2011; Grossman et al. 2011; Potter et al. 2010; Potter et al. 2003; Russell et al. 1993). This topic has been extensively discussed within the discourse of reproductive politics, often revolving around the framework of clinic-based versus the OTC access framework. Traditional discourse has highlighted various concerns regarding contraceptive pill usage, including sexual activities, religion, potential side effects, unsafe use, and political considerations (Hirayama 2022; Williams 2016; Dougall 2015; Kim 2013; Nguyen 2006; Matsumoto 2005; Vitale 2005; Norgren 2001).

More recent developments represent a departure from traditional discourse. A global-scale study investigating access to oral contraceptives has mapped its availability without a prescription in numerous countries (Grindlay et al. 2013). Updated guidelines from the World Health Organization (WHO) have introduced new recommendations advocating for the availability of oral contraceptives without the need for a prescription (WHO 2019a). Notably, during the pandemic in the UK, oral contraceptives, which were previously available only by prescription, were made accessible as a result of stakeholder involvement, including individual patients and patient groups (MHRA 2021a; MHRA 2021b). Most recently, the U.S. Food and Drug Administration (FDA) approved progestin-only to be available OTC (FDA 2023). The trend of improving contraceptive access and relaxing regulations remains contentious. However, there are growing efforts from various healthcare sectors, including patients, healthcare professionals, academia, and policymakers, who consider patients’ rights and prioritize patient and public involvement, patient-centric care, and sexual as well as reproductive health and rights activities (Barragués Fernández 2020; García-Martín et al. 2020; Pepper 2018).

In contrast, Japan follows a distinct approach, with contraceptives accessible exclusively through prescriptions. Guidelines established by the Japan Society of Obstetrics and Gynecology (JSOG) primarily address contraceptive use from a clinical perspective. These guidelines are centered on elucidating the effects and managing risks during clinical use, as delineated in 62 clinical question-based manuals. Clinical question 1 within these guidelines underscores that publicly funded national health insurance does not cover contraceptives prescribed for contraceptive purposes (JSOG and JMWH 2021). An in-depth exploration of the factors contributing to the unfavorable status of oral contraceptives in Japan has been undertaken in historical and social studies (Hirayama 2022; Matsumoto 2005; Vitale 2005; Norgren 2001). Several of these studies have aimed to identify the reasons for the challenging environment for oral contraceptives in Japan. They have scrutinized institutional requirements, such as the legalization of abortion in 1948, which preceded the approval of oral contraceptives in 1999 (Norgren 2001). Additionally, they have analyzed the interests of obstetricians and family planning stakeholders (Matsumoto 2005).

The historical context explains why the implementation of oral contraceptives in Japan was relatively delayed compared to other countries. Meanwhile, recent social initiatives related to women’s reproductive health and rights have emerged. Particularly noteworthy is the social and political engagement among non-profit and non-governmental organizations in Japanese civil society, focusing on disseminating sexual and health knowledge, along with policy recommendations. For instance, PILCON (pilcon.org) focuses on initiatives for the younger population, including sexual education and OTC availability advocacy for emergency contraceptives, while SOSHIREN (soshiren.org) has been raising awareness about oral abortion pills and advocating for the abortion law abolition. This trend aligns with the social movements of reproductive justice organizations, particularly in terms of self-determination (Cadena et al. 2022). However, as per the recent WHO contraceptive report, Japan continues to exhibit a trend of male-centric contraception through condoms (34.9%), with the usage of oral contraceptives (2.9%) being relatively low (WHO 2019b). A few years before the approval of the abortion pill in 2023, controversy arose over suggestions to set the price at around 100,000 yen (appx. 676 dollars, including a doctor’s consultation fee and hospital fees), emphasizing the significant difference in cost compared to prices set by United Nations Population Fund—roughly 6.55 dollars per tablet (Tsukahara 2023).

In light of the broader reproductive health and rights context, particularly in terms of access to medicines, it becomes evident that the challenge of obtaining oral contraceptives remains a significant concern for patients and users in Japan. A recent review of pharmaceutical regulations in various countries to reclassify oral contraceptives as OTC medicines underscores the importance of addressing this issue from a global health perspective (Ammerdorffer et al. 2022). Nonetheless, discussions within the JSOG guidelines and research outcomes have been confined to the domestic context. Furthermore, most studies have focused primarily on the use of oral contraceptives for contraceptive purposes. Therefore, there is a need to examine this topic from a transnational perspective, without restricting its utilization solely to contraceptive purposes, to derive a novel context around Japanese women who obtain and take the pill. To date, limited information is available on the patient experiences and perceptions of Japanese women regarding their use of overseas healthcare in East Asia. While all types of contraceptives in Japan require a prescription, South Korea has allowed OTC access to these medications since 1968 (Kim 2020). Second- and third-generation contraceptives are readily accessible in Korean pharmacies, whereas fourth-generation options currently require prescriptions from clinics.

Given the contrasting classification of oral contraceptives in the two countries, this study aimed to investigate the experiences of Japanese women concerning transnational health and self-care. More precisely, our focus rested on their use of oral contraceptives during extended stays in South Korea. Our goal was to explore the significance of transnational experiences reflected in perceptions of oral contraceptives and access. Concurrently, we aimed to understand the significance of the narratives and perspectives articulated by these women based on their experiences. This endeavor aimed to shed light on the worth of incorporating these insights into the improvement of prevailing clinical practices and healthcare systems.

## Methods

This study employed a qualitative research approach utilizing semi-structured interviews. The aim was to understand the experiences, perceptions, and contextual backgrounds of individuals who take oral contraceptives and/or engage in transnational health-seeking practices.

### Recruitment and Consent to Participate

Our study participants met three specific criteria: (1) adult Japanese women aged 20 years or older; (2) resided in South Korea since 2010 for mid- to long-term periods (defined as six months or longer) for reasons such as academic pursuits, employment, marriage, or other factors; and (3) had experience with oral contraceptives, including OTC access, commonly referred to as low-dose pills (低用量ピル, *teiyouryou-piru* in the Japanese context), while living in South Korea.

We employed two methods for participant recruitment for this study. Firstly, we utilized an online bulletin board with a language education center affiliated with one of the authors’ overseas research institutions. This approach was chosen to ensure a diverse sample of participants from various age groups and backgrounds. Secondly, participants were encouraged to refer potential participants through a snowball sampling method. Given the challenges of recruiting individuals with experience in transnational health and self-care, this approach was deemed suitable. Before conducting the interviews, we communicated with potential participants through email**.** We provided them with detailed information about the study, including consent forms, information sheets, withdrawal forms detailing the procedure to do so, and pre-interview questionnaires. The information sheets, in plain Japanese, covered the study significance and purpose, methodology, selection criteria, withdrawal procedures, risks and benefits, results dissemination, data disclosure, and personal information management for enquiries about the study. A concise summary of the information sheets was verbally provided to participants before the study began, with a reminder that they could withdraw at any time. They were then asked to confirm their understanding of the document. Interviews commenced once participants had reviewed and signed the consent forms and completed the pre-interview questionnaires. Participants received a cash incentive of 3000 yen or a gift certificate of equivalent value upon completing the interview, with the exception of one pilot participant. This study received ethical approval from the Ethics Committee of the Ethics Committee of Osaka University Hospital (approval number: 21089-4).

### Data Construction

To capture the participants’ experiences and perceptions, this study employed a semi-structured interview approach. The interview consisted of both retrospective and prospective questions. Retrospective questions aimed to elicit participants’ reflections on their experiences with and acquisition of contraceptive pills in both Japan and South Korea. In contrast, prospective questions focused on participants’ attitudes, perceptions of accessibility, and future expectations regarding oral contraceptive use (see Table 1). Furthermore, a pre-interview questionnaire was administered to facilitate the data collection for the interviewer. Female research assistants could be made available upon request by participants, as the lead investigator of this study was male. However, none of the participants opted for this assistance.

**Table 1:** Examples taken from the semi-structured interview guide

Retrospective question	Prospective question
What led you to start taking pills initially?	Are you planning to continue using pills in the future?
How did you go about getting pills in Japan and/or South Korea?	Could you share your preferences when it comes to accessing pills?
Did your reasons for taking pills change after you went abroad?	Could you please elaborate on (i) the factors influencing your choices in the pre-survey and (ii) the reasons behind your rankings?
What were the expenses associated with pills in Japan and/or South Korea? Were they covered by health insurance?	Do you have any opinions or requests regarding how pills are obtained in Japan?
How did you gain knowledge about pills in Japan and/or South Korea (e.g., through media, individuals)?	Do you have any messages for healthcare providers, policymakers, or anyone involved in healthcare regarding the use of pills?
Did you encounter any positive experiences, concerns, or challenges related to taking pills in Japan and/or South Korea?	How was the interview experience for you as someone who has prior experience with pills?

An efficacy check was conducted in August 2021, through a pilot interview to assess the effectiveness of the interview questions and the feasibility of achieving the research objectives. Formal semi-structured interviews were conducted from September 2021 to February 2022. It is important to note that this study’s primary objective was to understand the backgrounds of participants who have engaged transnational health-seeking and the contextual factors influencing their experiences. Therefore, data saturation was not pursued as a specific goal. Recruitment concluded after no further contact could be established with potential participants following the inclusion of the tenth participant. The interviews were conducted by utilizing both online and offline formats, with all participants expressing a preference for online interviews conducted through Zoom or Line apps. While the interviews were primarily conducted in Japanese, some Korean was utilized when necessary. This was particularly relevant when discussing the names of pills available in South Korea, communicating with local healthcare providers, and addressing cultural nuances. The one-on-one interviews, which typically lasted approximately 60–90 min, were recorded with the participants’ consent and transcribed verbatim.

### Data Analysis

The interview data underwent thematic analysis (Braun and Clarke 2006) using the QSR NVivo software. The analysis process consisted of two main phases. In the first phase, open coding was conducted. These codes encompassed significant words, sentences, and paragraphs extracted from the interview data. In the second phase, similar codes were grouped, forming higher-order categories. These categories were created based on the relationships and connections observed between the codes, as well as their concepts and meanings. Subsequently, in the second phase of analysis, overarching themes were identified, driven by the relationships and associations identified between the codes, concepts, and meanings. These themes were then sorted into thematic categories. One author conducted both the first and second phases of coding, and the appropriateness of the generated themes and thematic categories was verified through meetings and discussions involving both authors.

## Results

### Characteristics of the Participants

Eleven participants, including one in the pilot interview, participated in this study as either patients or individuals who regularly took contraceptive pills. On average, they had been residing in South Korea for approximately two and a half years. As exhibited in Table 2, most participants had prior experience obtaining and using both OTC pills in South Korea and prescribed pills in Japan. However, participants 8 and 9 did not conform to this typical pattern. Participant 8 obtained OTC pills directly from a South Korean pharmacy and had no prior experience with pills in Japan ( ×). Conversely, participant 9, despite being aware of the OTC access in South Korea and receiving a doctor’s recommendation to utilize non-prescribed pills for symptom improvement, opted for prescribed pills due to personal anxiety (#).

**Table 2:** Characteristics of the participants

	Occup	Address	Age	Repro. exp	Stay	Init. age	Purpose	Rx JP	OTC KR
P1	Unemployed, homemaker	JP	20 s	×	1 yr 6 mo	20 s	Contraception, adjusting menstrual cycle, dealing with skin troubles (acne)	✓	✓
P2	Student	KR	40 s	×	3 yr	30 s	Contraception, adjusting menstrual cycle, dealing with skin troubles (acne)	✓	✓
P3	Hairstylist	JP	20 s	×	1 yr 7 mo	10 s	Irregular menstruation	✓	✓
P4	Translator	KR	20 s	✓ in KR	4 yr 10 mo	20 s	Dealing with PMS (premenstrual syndrome)	✓	✓
P5	Office worker	JP	20 s	×	4 yr	20 s	Contraception, adjusting menstrual cycle, dealing with menstrual pain, dealing with PMS, dealing with skin troubles (acne)	✓	✓
P6	Instructor	KR	40 s	✓ in JP	1 yr 10 mo	20 s	Others (hormonal balance adjustment, anovulatory menstruation)	✓	✓
P7	Student, part-time job	KR	30 s	×	1 yr 2 mo	30 s	Adjusting menstrual cycle, dealing with menstrual pain, dealing with PMS	✓	✓
P8	Student	KR	20 s	×	1 yr 5 mo	20 s	Contraception, dealing with menstrual pain, dealing with PMS	×	✓
P9	Unemployed, homemaker	KR	20 s	✓ in KR	6 yr 4 mo	20 s	Adjusting menstrual cycle, others (treatment for polycystic ovary syndrome)	✓	#
P10	Student	KR	20 s	×	1 yr 6 mo	20 s	Adjusting menstrual cycle, dealing with skin troubles (acne)	✓	✓
P11	Journalist	KR	20 s	✓ in JP	1 yr 6 mo	20 s	Contraception, adjusting menstrual cycle, dealing with PMS, dealing with skin troubles (acne)	✓	✓

P with number, participant; *Occup.*, occupation; *Repro. exp.*, reproductive experience; *Init. age*, initial age; *Rx JP*, prescription access in Japan; *OTC KR*, over-the-counter access in South Korea.

### Thematic Overview

Participants provided detailed descriptions of their experiences with the use of oral contraceptives, from acquisition to consumption, from the individuals’ perspectives in both countries. This study’s thematic analysis uncovered three main categories with eight subthemes (Table 3).

**Table 3:** Results of thematic analysis

Three thematic categories and eight themes
Category 1 Experiences and perceptions of obtaining and taking pills, including OTC access
Theme 1 Convenient access to OTC pills supports daily health and self-care
Theme 2 Facilitating pill use through friends, relatives, advertisements, and the Internet
Theme 3 Challenges in accessing pills through clinics, pharmacies, and online sources
Category 2 Individual and social perceptions of pills and their access in Japan among actual users
Theme 4 *Shikiiga-takai*: cost, distance, psychological, and social barriers hinder pill access in Japan
Theme 5 Decisions on health insurance coverage based on treatment or contraception feel like *kotoba-asobi*
Theme 6 Social perceptions of pills and their connection to abortion issues in Japan
Category 3 Enhancing pill access: transnational health and self-care experiences and opinions
Theme 7 The “open” social image of using pills in South Korea among Japanese women and “global” insights
Theme 8 Transnational health-seeking: diverse access and the significance of choice and rights

The participants found low-dose OTC pills in South Korea to be easily accessible, convenient, and affordable. They highlighted that these pills empowered them to manage their health, covering treatment, contraception, and daily care effectively. In contrast, participants described their healthcare experiences in Japan as having a high threshold using the term *shikiiga-takai* (敷居が高い) to convey this sentiment. They associated this perception with a sense of safety owing to rigorous medical interventions involving thorough examinations and diagnoses. Challenges related to acquiring and using contraceptive pills in Japan were also discussed. These include financial burdens, reluctance to visit gynecological clinics, social stigma, and misconceptions about issues like sexual promiscuity or infertility. Furthermore, the participants shared their perceptions of oral contraceptives, strategies for pill usage upon returning to Japan, and the limited access to prescribed pills in the country.

#### Category 1 Experiences and Perceptions of Obtaining and Taking Pills, including OTC Access

This thematic category included three themes about the participants’ experiences and how they felt about the process of obtaining and taking contraceptive pills.

#### Theme 1 Convenient Access to OTC Pills Supports Daily Health and Self-care

The majority of participants discussed OTC oral contraceptives compared to prescription-based access. They highlighted the convenience of OTC access in the local context, noting its affordability and the ease of quickly obtaining the pills from any pharmacy. An example of this is provided below:

Ultimately, the biggest factor for me is the convenience of being able to buy it easily. Plus, the fact that it’s not too expensive is also important. I’m still a student studying abroad, so I don’t have a lot of money, and that plays a significant role. (Participant 8, 20s, student, KR)

Consequently, for many of the participants who had integrated contraceptive pills into their daily routines, these pills were perceived to be very familiar and akin to common medications like “cold remedies” or “vitamins supplements.” An example of this is provided below:

Somehow, I feel more satisfied with it in Korea because you can buy it anywhere, it’s really affordable, and you can manage your own health. So, I find it convenient. (Participant 5, 20s, office worker, JP)

And since you can buy it quite easily without much embarrassment, just like saying, ‘Can I have some cold medicine?’ It doesn’t come with any significant mental burden. (Participant 4, 20s, translator, KR)

The advantages of this convenience were further underscored as the participants shared their experiences in situations where healthcare accessibility was relatively limited owing to different factors, such as emigration, resulting in the discontinuation of their home country’s national health insurance or restriction of enrolment for an equivalent insurance package in the host country. This necessitates the availability of OTC medications. An example of this is provided below:

But in Korea, you don’t need health insurance [to obtain pills], and you can get them at the pharmacy. It was super convenient. Even for me without any [public] health insurance I could get it properly, so I thought it was very rational at that time. (Participant 1, 20s, unemployed, JP)

#### Theme 2 Facilitating Pill Use through Friends, Relatives, Advertisements, and the Internet

During the interviews, the participants explained how they acquired information on locally available oral contraceptives and what prompted them to use these pills. The majority mentioned that they had learned about the pill through conducting searches on the Internet in Japanese. These included searching in blogs. An example of this is provided below:

There was a blog written by a Japanese-Korean couple, and the person in the blog was taking Marvelon too, and I thought, ‘Oh, we’re taking the same thing!’ They mentioned that there’s a similar product in Korea called Mercilon. (Participant 2, 40s, student, KR)

Some participants became aware of the local methods for obtaining contraceptive pills through friends with the same experiences during their language studies in the region. In some unique cases, participants learned about the availability of OTC access to pills when they traveled and encountered pill advertisements. Additionally, a few participants noted that discussions about contraceptive pill advertisements were topics of interest within the Japanese expatriate communities. An example of this is provided below:

When I went to Korea [for a trip], they were advertising it on TV commercials and stuff, so at that time, I was quite surprised. It also became a topic among Japanese wives living in Korea. (Participant 4, 20s, translator, KR)

Furthermore, some participants stated that they had never attempted to acquire knowledge through official health information sources, as they found these sources less user-friendly. This emphasizes the importance of unofficial health information sources, such as blogs, as the primary source of health-related knowledge. An example of this is provided below:

I don’t recall ever coming across anything written by Koroshou [厚労省, Ministry of Health, Labour and Welfare] or the like that was easy to understand... As a college student back then, I couldn’t imagine myself taking an interest in the Koroshou website. (Participant 1, 20s, unemployed, JP)

#### Theme 3 Challenges in Accessing Pills through Clinics, Pharmacies, and Online Sources

When describing obtaining and using pills from clinics or pharmacies, the participants seldom reported complete satisfaction. Concerns and difficulties were identified when obtaining and using oral contraceptives in the absence of clinical intervention or in foreign countries. For instance, some participants encountered challenges in identifying the equivalents of the pills they had used in Japan to those available in South Korea, with questions such as *which one resembles Yaz?* (participant 7, 30 s, student, KR). They also experienced difficulties communicating with the local healthcare providers. However, in most cases, when language barriers were encountered during pharmacy visits, participants initially overcame these challenges by seeking assistance from friends proficient in the local language or by actively utilizing translation applications. An example of this is provided below:

Well, you know, in Japanese, I can read and understand all the side effects and precautions, but in Korean... It’s a bit challenging. If I find the numbers [on the pill package], I can probably figure out how many times a day, but I’m often lost when it comes to other precautions. So, every time, I take pictures and use Papago for image translation. (Participant 10, 20s, student, KR)

Furthermore, participants expressed apprehension about delayed deliveries or receiving counterfeit pills when making use of online purchasing platforms. An example of this is provided below:

I think it’s nice that you can get low-dose pills online, but personally, I’m a bit skeptical about buying things online in Japan, because there are also things like receiving counterfeit products. So, I feel it might be better to buy them from a pharmacy or a pharmacist. (Participant 5, 20s, office worker, JP)

However, even in cases involving clinical interventions, some participants reported that they experienced difficulties with using the pill. For instance, certain participants experienced side effects or encountered challenges while taking prescription pills in the clinic. Interestingly, some participants found that OTC contraceptive pills were free of side effects and compatible with their bodies. Additionally, there were instances in which participants had already acquired contraceptive pills locally but received no acknowledgment or support from Japanese clinics; in some cases, they faced moral blame from healthcare providers (also linked to Theme 7). An example of this is provided below:

So, when I’m taking pills that aren’t available in Japan, the doctor treated me really coldly, as if they couldn’t guarantee anything if something were to happen in Japan. That’s how he/she said things like that. I mean, if that were the case, the ones [I’m using] might be consumed more [by women worldwide] than Faboir [in Japan], you know? (Participant 1, 20s, unemployed, JP)

#### Category 2 Individual and Social Perceptions of Pills and their Access in Japan among Actual Users

This thematic category encompasses the specific thoughts and feelings of actual individuals who use these pills, have experienced two or more types of access to the pills, and offer insights into the accessibility of Japan’s contraceptive pill and related societal issues. This category has been organized into three main themes.

#### Theme 4 Shikiiga-takai: Cost, Distance, Psychological, and Social Barriers Hinder Pill Access in Japan

Participants retrospectively discussed both the direct and indirect challenges associated with obtaining contraceptive pills in their home country. These obstacles were related to established medical practices and concerns or negative perceptions of individuals who utilize these contraceptive pills within their immediate surroundings and society, including misconceptions about oral contraceptives. An example of this is provided below:

People tend to think that I’m taking them for contraception (Participant 3, 20s, hairstylist, JP).

Maybe people around me will be put off by it [pill-taking]? They might say things like, ‘Oh, she is taking pills’ (Participant 9, 20s, unemployed, KR)

But in the past, if you were taking them, people would sometimes say things like, ‘Don’t you want to get pregnant?’ So, I’ve been asked things like, ‘You’re not having a second child?’ quite a bit. But in my case, I was taking them for treatment. (Participant 6, 40s, instructor, KR)

Some reported that they had not learned much about oral contraceptives, while others mentioned that sex education was insufficient even when it was available. This situation is metaphorically described as *shikiiga-takai*, which is a common Japanese idiom meaning “high threshold.” Regarding these challenges, most participants recounted undergoing various medical examinations, such as inserting hands or instruments into their vaginas for a test called *naishin* (内診), pelvic examinations, and ultrasound tests, before starting to utilize the pill. Instances of receiving pills based solely on history-taking are rare. Factors contributing to this high threshold include the financial and time costs associated with visiting a clinic for examination and the need for regular follow-up visits.

Furthermore, psychological and sociocultural factors were emphasized. One aspect involved the psychological difficulty of visiting a gynecologist, often associated with distorted perceptions of young female visitors in clinic waiting rooms (related to Theme 6). Another aspect related to sociocultural factors where contraceptive pill use was associated with either giving up on childbearing or sexual promiscuity, as one participant noted: *also, there’s a lot of discrimination like saying, ‘If someone is taking pills, that person must be doing sex work’* (participant 5, 20 s, office worker, JP). Differences in the perceptions of the utilization of contraceptive pills between women who had already experienced childbirth and previous generations of women were also noted as contributing factors to this high threshold. Additionally, some participants mentioned the historical fact that oral contraceptives took a considerable amount of time to gain official approval and authorization in Japan compared to other countries, further contributing to the notion of *shikiiga-takai*. An example of this is provided below:

In the case of Japan, it was slow to get approval, and there are fewer people, even celebrities, who openly talk about it with regard to obstetrics and gynecology, I think. (Participant 6, 40s, instructor, KR)

#### Theme 5 Decisions on Health Insurance Coverage based on Treatment or Contraception Feel like Kotoba-asobi

All participants reported using this medication in various situations and contexts, ranging from symptom improvement to contraception, as well as for multiple purposes, with some initially taking it for one specific reason in the clinic but later altering their utilization intentions based on the observed secondary effects. An example of this is provided below:

I started taking it about six months after beginning, mainly as a measure against menstrual pain and PMS. Now, after continuing it, probably due to factors like age, I’ve completely eliminated skin problems. (Participant 5, 20s, office worker, JP)

Because it can also provide contraception, allow you to plan your menstrual schedule, and help with... well, things like skin issues, which I didn’t really understand, and of course, I don’t know much about menstruation-related stuff like PHS [PMS], but there are various benefits that can be done to ease those symptoms from such a small pill. (Participant 2, 40s, student, KR)

In fact, in Japan, eligibility for national health insurance coverage for this medication is limited when used for therapeutic purposes. Some participants viewed this situation as *kotoba-asobi* (言葉遊び)—a wordplay. An example of this is provided below:

What can I say? It’s just that even though it’s women who end up getting hurt, and there is also a preventive aspect to protect the body, the reason of ‘It’s not allowed because it’s for contraception purpose’ somehow doesn’t make sense to us women. (Participant 5, 20s, office worker, JP)

It kind of feels like kotoba-asobi, doesn’t it? Whether you just say it’s for contraception purposes or you say it’s because of severe menstrual symptoms or irregular periods, that’s where the difference lies, right? So, it seems a bit pointless, you know. (Participant 11, 20s, journalist, KR)

Some participants also expressed discomfort in the linguistic context where this medication was referred to as a contraceptive pill. Furthermore, participants individually attributed a wide array of existential meanings to this medication, such as “choice,” “amulet,” “peace of mind,” “magic pill,” “women’s rights,” and “therapeutic remedy.”

#### Theme 6 Social Perceptions of Pills and their Connection to Abortion Issues in Japan

Some participants expressed that Japan’s constant accessibility to contraceptive pills through prescriptions did not align with the pill’s patient/user-centered model. While they acknowledged the recently introduced remote medical services as a positive development, they questioned whether this improved accessibility truly benefited individuals using these pills. They further highlighted the reality of young Japanese students, who are menstruating or in need of contraception, as the cost and psychological barriers to accessing contraceptive pills remain significant. An example of this is provided below:

But for minors, who usually holds their insurance card? It’s their parents, right?... Even if they become an adult while in college, there are cases where the insurance card is issued by their father’s company... one might say [online prescription] has become more convenient, to be honest. But, within the context of the image of the pills [in Japan], when someone hears, ‘You can get pills online,’ can high school girls just go online at home and consult a gynecologist?... Prescriptions go to the home FAX... Your parents, maybe even your dad, might see it. The mail [as well]... When you’re receiving telemedicine, you might still end up on the phone. Even if you’re in the next room or your own room, it can be heard. If the parents ask, ‘Who were you talking to?’ (Participant 4, 20s, translator, KR)

Participants reported several societal factors contributing to these barriers, such as persistent cultural resistance to artificial methods, vague anxieties about the side effects, lack of contraceptive education, and a predominant focus on condom-based education. An example of this is provided below:

I do think there’s a culture that strongly resists new things. So, I think Japanese people have a strong dislike for pills, which are perceived as an artificial contraceptive. (Participant 5, 20s, office worker, JP)

In Japan, there is a perceived inverse relationship between contraceptive pills and condom use. However, participants believed that combining contraceptive pills and condoms was more effective and advocated a shift in this perception. An example of this is provided below:

When a man and a woman have such a relationship, and the woman says, ‘I’m on the pill,’ some men say, ‘Then I don’t have to wear a condom’ … I think it’s really selfish to do that, and I think it’s such a great justice to wear a condom and use both [to contraceptive and] to prevent sexually transmitted diseases. (Participant 1, 20s, unemployed, JP)

Notably, some participants perceived a close connection between the accessibility of contraceptive pills and abortion. They anticipated that the availability of OTC contraceptive pills would reduce the number of abortions in Japan. Furthermore, the participants observed a societal preference for abortion over contraception. However, they believed that the prevalent practices of abortion (generally known as *souhahou*—掻爬法—the suction method) and use of emergency contraceptive pills in Japan were relatively burdensome on the female body compared to the regular preventive use of contraceptive pills. As one participant put it: *I still have the impression that the low-dose pills are better than taking the morning-after pill* (participant 10, 20 s, student, KR).

#### Category 3 Enhancing Pill Access: Transnational Health and Self-care Experiences and Opinions

This thematic category comprised two themes related to participants’ transnational health management experiences concerning the availability of contraceptive pills, as revealed through interviews. These themes revolve around the perceptions and perspectives proposed based on transnational experiences to improve access to such pills.

#### Theme 7 The “Open” Social Image of Using Pills in South Korea among Japanese Women and “Global” Insights

This theme encompasses the transnational aspects of the Japanese participants’ experiences and perceptions on the access to contraceptive pills in South Korea. To them, contraceptive pills in South Korea appeared to be easily accessible and carried positive connotations, contrasting with the more discreet and secretive image of the same pills in Japan. As one participant noted: *Japanese people tend to be secretive, while Korean people have a relatively open image* (participant 9). Their experiences included encounters with advertising in the media that were commonplace. They also mentioned the cultural aspect of South Korea’s fast-paced *ppalli-ppalli* (빨리빨리, which means “chop-chop” in Korean) culture, where contraceptives were readily accessible and pertinent and could be obtained quickly and effortlessly. They positively perceived the accessibility of contraceptive pills and societal perceptions in South Korea, which is in stark contrast to the negative, concealed, and worrisome perception of the contraceptive pill in Japan. The only differences they noted were minor ones in the pills themselves, such as in packaging and color. Furthermore, they highlighted differences in pharmacy types, such as dispensing pharmacies and the process of receiving pills. An example of this is provided below:

In Japan, pills are often available only at pharmacies near or directly connected to obstetrics and gynecology clinics… so if they weren’t nearby, you had to order. That was a bit inconvenient. (Participant 6, 40s, instructor, KR)

Well, until now, Japanese pharmacies didn’t really have much demand beyond taking prescriptions, right? (Participant 11, 20s, journalist, KR)

Nonetheless, some participants’ perceptions regarding the accessibility of contraceptive pills extended beyond the differences between the two countries, to a much broader global perspective. For instance, one participant felt deeply secure knowing that the same brand of contraceptive pills had been used by countless women worldwide for a significant period of time and felt a profound sense of unfairness in the ethical dilemma she encountered during her visit to the clinic at that time (also related to Theme 3). Another participant described a situation as peculiar, in which this medication, which had long been used overseas and was officially regulated by the local pharmaceutical authorities, suddenly transformed into something dangerous (as a result of unchanging ingredients) upon entering Japan. Consequently, based on their transnational health management experiences, the issue regarding the accessibility of contraceptive pills in South Korea was perceived as a highly global issue as opposed to just being an internal issue. An example of this is provided below:

After all, I found out that there is a large number of women [worldwide] taking [the same brand] pills, so I was quite relieved in Korea [when using OTC pills] … At that time, despite being a doctor, I was quite disappointed with the way he/she handled it… My emotions were at their worst, and I became extremely nervous. But you know, the next day, it’s not like I forgot everything once I went to sleep. However, any prejudice against Mercilon didn’t arise just because of that… and it seems like I’ll continue taking it. It’s irrelevant to me, that doctor from back then. It might be in real trouble if something happens, but even more than the doctor’s threats, there are thousands of women taking Mercilon before me. (Participant 1, 20s, unemployed, JP)

The ingredients of medicines are all the same all over the world... I think some of the ingredients are stronger [middle-dose] or weaker [low-dose] in the combination. I don't think there is anything particularly dangerous about it... Even though there are many women in the world who are actually taking. (Participant 11, 20s, journalist, KR)

#### Theme 8 Transnational Health-Seeking: Diverse Access and the Significance of Choice and Rights

In contrast to the previous themes, this theme explores the responses related to participants’ interview questions in relation to their prospective perceptions of the accessibility of contraceptive pills. Participants conveyed their dissatisfaction with the current centralized prescription-based approach to the accessibility of contraceptive pills in Japan, primarily due to the associated high uninsured costs. Their experiences with transnational health-seeking had made them aware of the convenience of OTC options. Before the interviews, participants were surveyed to identify their top three preferred access methods (see Table 4). These preferences were elaborated on in detail.

**Table 4:**
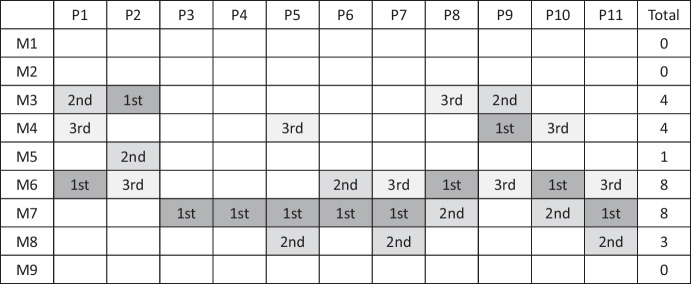
Results of the pre-survey on participants’ desired pill access

For the pre-survey, we presented nine models of access to oral contraceptives. Participants could choose up to three preferred models and rank them from first to third preference. Three types of carbonized colors (grey) were placed after the ordinal notation to visually represent their positions. Some participants chose only one or two options. Here, ‘M’ stands for the acronym for each model. M7 received the most votes for first place, while M6 received most votes for second and third places.

M1: Receive low-dose pills through out-of-pocket clinic visits.

M2: Obtain low-dose pills through online consultations in the case of out-of-pocket care, with delivery by mail.

M3: Utilize insurance-covered care for online consultations, obtain a prescription, and pick up the pills at a pharmacy.

M4: Access low-dose pills through insurance-covered clinic visits, obtain a prescription, and pick up the pills at a pharmacy.

M5: For the initial prescription, seek out-of-pocket care, get a prescription, and acquire the pills at a pharmacy. For subsequent refills, forego clinic visits and receive guidance from a pharmacist at the pharmacy before obtaining the pills.

M6: For the initial prescription, use insurance-covered care, get a prescription, and acquire the pills at a pharmacy. For subsequent refills, skip clinic visits and receive guidance from a pharmacist at the pharmacy before obtaining the pills.

M7: Obtain low-dose pills directly from a pharmacy without a prescription.

M8: Obtain low-dose pills directly from a pharmacy or online without a prescription.

M9: Please provide any other original suggestions, for example, “Make them all available at no cost,” etc.

Participants exhibited two distinct primary preferences for accessing contraceptive pills, with a clear order of preference. The first preference involved initiating the process with a clinic visit, where suitability was assessed through insurance consultations, followed by obtaining the prescription-based pills. The second preference was to start with OTC contraceptives from a pharmacy beginning with the second cycle of medication, assuming that no issues arose with the initial prescription-based pills. However, even for those who had progressed beyond their second cycle of medication, some participants acknowledged the existence of anxiety and the need for health counseling. They believed that individuals in need of such support should visit clinics for prescription-based access, while those who did not require such assistance should have the option to obtain OTC contraceptives from any pharmacy. This approach was perceived as the ideal one. Nevertheless, it was emphasized that within pharmacies or among pharmacists, their role should extend beyond merely dispensing medication; they should also provide effective medication guidance. An example of this is provided below:

For me, it would be best if you could buy it at a pharmacy like in Korea, but not by simply handing it over after asking them to ‘give it to me.’ Just one time the pharmacist asked me things like ‘Is this your first time?’ and ‘Did you have any symptoms?’ in Korea. You can buy it at a pharmacy, but I think it would be best for both of us if we asked questions like that. (Participant 8, 20s, student, KR)

The participants continued cross-border efforts to obtain contraceptive pills, such as purchasing bundled medication at local pharmacies or coordinating international shipments through their local family members or acquaintances. This underscores the need for alternative strategies to access contraceptive pills beyond national boundaries. These alternatives were categorized into six types (see Table 5) based on the participants’ preferences and actual strategies for potential scenarios in the event that prescription-based access was restricted once again. One aspect to be considered was that participants’ bundle purchasing with consideration of the expiry date in this context resembled the concept of just-in-time buying in terms of the conditions of the contraceptive pills.

**Table 5:** Types of transnational health-seeking behaviors when participants are confined to prescription medicines

Transnational alternative health and self-care strategies
1. Making bundle purchases at local pharmacies
2. Using international postal services to have medication sent by local family members or acquaintances or bringing it with them upon return
3. Using e-commerce websites (in Japanese)
4. Visiting hospitals (traditional and orthodox access)
5. Discontinuing medication altogether
6. Continuously taking medication in a hybrid manner, combining e-commerce, OTC, prescription, and discontinuation

However, it is important to note that participants who had experience obtaining oral contraceptives through online platforms expressed a combination of positive sentiments regarding convenience and affordability, along with concerns about delayed delivery and being provided counterfeit medication. Nonetheless, their choice to acquire pills through online platforms was significantly influenced by their familiarity with a particular global brand that they had previously obtained through OTC services during their mid- to long-term stay in South Korea. This familiarity reduced anxiety, fostering a sense of commonality among women worldwide who were also utilizing the same pills, therefore reassuring them. In conclusion, participants perceived the accessibility of contraceptive pills as being diverse and affordable, ultimately recognizing the issue as a multifaceted choice and a matter of human rights. An example of this is provided below:

Actually, it’s quite remarkable that Japanese women aren’t given the right to protect their own bodies or anything like that. I think it might be a significant violation of human rights, from a woman’s perspective. (Participant 1, 20s, unemployed, JP)

Somehow, it would be great if people who want to go can go to the obstetrics and gynecology clinics to get it, and those who don’t really care can just buy it at a regular pharmacy, having the option for both is appreciated. (Participant 5, 20s, office worker, JP)

## Discussion

This study explored the narratives of Japanese women with a background of utilizing OTC contraceptive pills in South Korea and identified the following key findings, namely (1) some participants perceived contraceptive pills as a global product, considering them to be common medications among women worldwide; (2) a few participants encountered difficulties when discussing the use of contraceptive pills purchased in South Korea with doctors at clinics in Japan upon returning home; (3) and most participants continued using pills by leveraging transnational opportunities. These included procuring an adequate supply of contraceptive pills from South Korean pharmacies through just-in-time purchases, requesting that they be sent by family or acquaintances after sourcing them from online retailers or opting to discontinue using contraceptive pills altogether. The participants’ perspectives, shaped by their experiences in transnational health and self-care, confirmed their roles as experts who experienced both healthcare systems.

From the participants’ responses, the advantages and disadvantages of accessing various types of contraceptives were identified. These findings align closely with those of previous studies, indicating that the use of OTC contraceptive pills by participants is associated with higher rates of continuous and limited contraindicated utilization (Kennedy et al. 2019). Furthermore, participants’ perceptions of the accessibility of oral contraceptives and transnational health-related behavioral patterns are largely consistent with prior research, which implies that OTC options are likely to be considered if oral contraceptives are available at affordable prices (Potter et al. 2011). Participants expressed support for OTC access as well as the option to visit a clinic, underlining the necessity to improve access to health checkups (White et al. 2013), particularly when new patients were concerned about potential side effects that would arise from taking contraceptive pills, rather than patients with more experience with oral contraceptives.

Conversely, various challenges that hinder oral contraceptive access were observed. Language barriers have been emphasized in the healthcare utilization of migrants in host countries with recommended support measures (Keller and Alishio-Caballero 2021). Participants in this study initially experienced language barriers during the early stages of migration but demonstrated a proactive approach to overcome them by utilizing translation apps, being accompanied by local acquaintances, or actively seeking medication counseling. These approaches differ from conventional approaches in that they address language barriers. Moreover, questions have been raised about the effectiveness of telemedicine improvements, such as online prescriptions, implying that these advancements do not consistently yield positive outcomes. These challenges reflect the fact that fully guaranteeing the accessibility of oral contraceptives is challenging, emphasizing the ongoing obstacles in this area.

Some participants expressed experiencing transnational health-seeking challenges when receiving prescriptions from their doctors. These challenges include physicians’ aggressive attitudes and inadequate responses to the participant’s health concerns, especially when obtaining new prescriptions for oral contraceptives after having finished the OTC pills obtained abroad upon returning to their home country. This hindered effective communication between patients and healthcare providers. Participants perceived the contraceptive pills they had officially purchased from local pharmacies as OTC medicines and considered them essentially identical to their global perspectives. This finding resonates with the perception that “public health is global” (Sawleshwarkar et al. 2020). In contrast, physicians categorize contraceptive pills as domestic pharmaceuticals and perceive transnational patients as informal and hazardous. Here, a dilemma in communication arose from the difference in the perception of contraceptive pill-taking routes between the two.

Notably, similar situations of prejudice in clinical settings and healthcare systems have been observed in patient treatment, such as racial and gender minorities (Okoro et al. 2020; Cuevas et al. 2016; Sabin et al. 2015). As a solution to clinical ethics, we propose that the healthcare field should be reminded or guided by the principle of avoiding value judgments when addressing the health concerns of patients who experience transnational contexts. This implies that the time spent in clinical settings should be devoted to making genuine assessments and providing the necessary treatment for patients’ symptoms and illnesses, as opposed to being influenced by value judgments stemming from differences in patients’ attitudes and perceptions of utilizing contraceptive pills.

Considering the generously shared participants’ insights, this extended beyond mere opinions on transnational health-seeking behaviors. It evolved into a critical framework that was perceived and assessed by individuals, encompassing not only domestic healthcare challenges but also positioning their transnational health and self-care in the context of utilizing the contraceptive pill and existential understanding of the contraceptive pill itself on a global scale. This reflective dimension closely coincides with the critical medical anthropological perspective, characterized by blending politico-economic considerations with a culturally sensitive analysis of human behavior based on anthropological methods (Witeska-Młynarczyk 2015). Their global insights into access to contraceptives and the pills themselves were shaped during mid- to long-term stays, with experiences in South Korea during their adulthood and in Japan during their younger years. This temporal distinction allows for a comparison of experiences and perceptions between the two countries. Through their transnational lens, attitudes toward contraceptive pills and global acquisition strategies were developed based on firsthand experiences as actual users and/or patients.

Their lens provides a nuanced perspective, moving beyond vague opinions, such as those using terms like “promiscuous” or “obscene,” which can pigeonhole individuals (i.e., social stigma) and carry strong negative connotations regarding access and pill-taking. Building upon this foundation, we underscore their transnational health-seeking behavior, globalizing their approach to taking pills as an integral aspect of maintaining their well-being and self-care routines in their everyday life. This also entails the possibility that migrant Japanese individuals, upon returning home, might discontinue medication owing to *shikiiga-takai* (Theme 4) in Japan. This discontinuation could occur if they experienced a shift back to prescription-only access, whether briefly or indefinitely. In connection to the transnationality of the participants in this study, a recent review on transnational health examined the literature with a focus on the research subjects, specifically targeting migrants and those who remain behind (e.g., parents, children, and friends) in the country of origin (Roosen et al. 2021). Our study further expanded the scope of transnational users and/or patients by investigating Japanese women who, after migrating to South Korea, returned to Japan, either temporarily or permanently, revealing the presence of transnational Japanese patients in Japan.

By focusing on this globalized pill-taking context, the participants have provided valuable insights into the distinctive aspects of transnational dynamics in their health and self-care experiences. Particularly noteworthy are the participants’ ideas about the “choices” and “fusion” aspects of access to healthcare, in connection to the rarely explored concept of medical pluralism in the context of oral contraceptive access. This concept can be described as advocating for the existence and utilization of one or more alternative healthcare systems, including the use of non-prescribed medicines (Wade et al. 2008). Recognizing medical pluralism as a matter of justice in health policy is crucial for supporting patients’ autonomy in choosing non-harmful medicines, including oral contraceptives, and acknowledging patients’ health and human rights (Muyskens 2023). Participants’ insights derived from their transnational health and self-care experiences have the potential to serve as valuable resources in shaping healthcare policies. From this perspective, we derived two distinct models to improve access to oral contraceptives in Japan.

Transitioning from department-centric to patient-centered access (model 1): In most cases, oral contraceptives can only be obtained from obstetrics and gynecology clinics. There are possible reasons for this, such as a paternalistic approach in clinical settings aimed at managing the risk of side effects and abuse issues associated with the use of contraceptive pills in the existing system (Sorano et al. 2021; Martin 2013). However, from a patient-centered perspective, it can be seen that the provision of oral contraceptives operates with a primary focus on the institutional systems of existing medical departments, such as obstetrics and gynecology as well as internal medicine, rather than a focus on the patients themselves. While there have been some cases illustrating certain cultural and functional aspects of paternalism in healthcare (Thompson et al. 2022; Murgic et al. 2015), the findings of this research instead imply that situations resembling medical paternalism may pose challenges to the medical accessibility of patients and end-users. Therefore, it is essential to expand the scope of provision for oral contraceptives beyond current systems that are limited to obstetrics and gynecology. Creating a way for patients to seek medical advice and receive prescriptions for oral contraceptives from experts in other relevant fields (e.g., dermatology, pediatrics and adolescent medicine, family medicine, and psychiatry) would deconstruct the current paternalistic systems that limit access to oral contraceptives and thus strengthen the quality of patient health and care.

Aiming for a change to the OTC approach (model 2): When considering the change in oral contraceptives from prescription-based to OTC status, it is important to approach this from a multipurpose standpoint beyond merely positioning them as contraceptives, as seen in most previous research. Given that Japan already has a health policy resource for pharmaceuticals requiring guidance (要指導医薬品), which is part of its OTC medicines access system and a measure that can be considered before the complete transition to OTC, this approach is worth considering for policy implementation as an option. For this to occur, expanding the use of electronic health information tools already in use such as the My Number personal ID card (マイナンバーカード) and medication notebook (お薬手帳, personal health record), during pharmacy screenings and similar occasions can be considered. These tools could aid pharmacists and related professionals in performing patient health screenings, thereby ensuring the appropriateness of OTC contraceptives for each individual. They could also provide accurate and convenient self-care resources for patients and users at little or no cost, which may elevate standards in patient safety and consultation. For the expansion of OTC operations, the potential involvement of registered salesclerks (登録販売者) that are qualified to sell non-prescription drugs could be considered. In the daily context of Japanese pharmacies and drug stores, where it is common for the pharmacists to be unavailable during late hours or weekends, registered salesclerks could provide pills to patients/users based on electronic health records.

The two suggestions, including the switch to OTC, should not be seen as an absolute transition from a prescription-limited situation but rather as a diversification of options or a hybrid access model based on research participants’ perceptions and medical pluralism. In fact, the two models discussed in this study consider policy resources to improve physical access to contraceptive pills, which is the gateway to obtaining them. Policies should be considered to enhance the accessibility of contraceptive pills, such as reducing costs for low-income women and girls through public insurance or vouchers and promoting women’s health and sexuality education. This is within the scope of sexual and reproductive health and rights, extending beyond clinical settings associated with contraception and healthcare. In this respect, the participants highlighted the challenges of acquiring information on the usage of oral contraceptives that includes both their effectiveness and potential side effects during adolescence. They also pointed out the intricacies of official health information in adulthood.

Thus, there is an unmet need to improve the provision of information, education, and counseling by improving the user experience through health education and online platforms. The point here is to embrace efforts to emphasize health promotion and information beyond traditional interventions by health authorities, particularly through social media platforms, which are predominantly used by the younger generations (Lim et al. 2022). Addressing these communication gaps or absences is a critical strategy to improve accessibility and awareness of current and future women’s health issues. Overall, these suggestions not only reflect improvements in oral contraceptive access but also present potential solutions to sexual and reproductive health and rights challenges in Japan. This might include reducing the number of abortions, addressing menstrual-related physical and mental symptoms faced by women in their daily lives, enhancing women’s quality of life, and promoting health equity for women, as perceived from the participants’ insights. This also implies the feasibility of implementing these suggested access models in the context of Japan and other countries and regions where access to prescription-only contraceptive pills is the norm.

This study has limitations, as it focused on a specific health context experienced by a small number of individuals. Therefore, it may not fully capture the diversity of experiences of broader transnational health-seeking patients and pill users. In particular, as our participants were adults, the experiences of adolescents were excluded, and the findings likely represent only a subset of the actual user population. Nevertheless, we gained insights into the past experiences of the participants during their adolescence as well as current barriers, including those related to telemedicine in Japan. Methodologically, author 1, who possesses a transnational background themself, led the data construction and coding, which potentially led to the emphasis of similar transnational aspects in the study. To mitigate this, numerous meetings were held regarding the data, codes, and initial themes to arrive at an agreement in data interpretation.

This study, therefore, highlights the need for further research on issues that are global and related to women’s health from the perspectives that participants genuinely care about, namely (1) participants’ hesitancy to obtain pills through online pharmaceutical retail sites; (2) knowledge and insights gained through non-standard sources, including blogs and social media, regarding the utilization, acquisition, and related issues of contraceptive pills; and (3) participants’ surprise at encountering uncommon OTC oral contraceptive advertisements during their everyday life abroad. This will require delving into the intricacies and particulars of these concealed health issues beneath the iceberg, considering the ethical, legal, and social aspects.

## Conclusion

The thematic analysis of the transnational health and self-care of Japanese women’s experiences in taking oral contraceptives, including OTC access in South Korea, revealed challenges in communication and health-seeking issues as a result of different understandings between transnational patients and healthcare professionals in acquiring contraceptive pills. In terms of participants’ attitudes and insights, this study captured critical medical anthropology aspects. Participants questioned the appropriateness of limiting access to contraceptive pills that have been prescribed for both visiting patients and vulnerable individuals within the existing healthcare system from a global perspective. Considering the current healthcare system in Japan and the context of transnational patients, we suggest two models for improving access to oral contraceptives. These models include expanding access to clinical contexts and transitioning to OTC medication. Future research should focus on unstructured health information sources, such as advertisements and blogs, which may play a role in shaping transnational health-seeking patterns, including international transmission through local networks and e-commercial websites.

## Data Availability

The author wishes to maintain the confidentiality of the research data in order to safeguard the individual privacy of the respondents. All pertinent information and resources are stored in compliance with the ethical protocols of the Ethical Review Board at Osaka University Hospital.
